# Yeast Cell wall Particle mediated Nanotube-RNA delivery system loaded with miR365 Antagomir for Post-traumatic Osteoarthritis Therapy via Oral Route

**DOI:** 10.7150/thno.46761

**Published:** 2020-07-09

**Authors:** Long Zhang, Hang Peng, Wan Zhang, Yankun Li, Liang Liu, Tongtong Leng

**Affiliations:** 1 Frontier Institute of Science and Technology, Xi'an Jiaotong University, Xi'an, Shaanxi 710054, PR China.; 2 Health Science Center of Xi'an Jiaotong University, Xi'an, Shaanxi 710061, PR China.; 3 Department of Orthopaedics, The First Affiliated Hospital of Xi'an Jiaotong University, Xi'an, Shaanxi 710061, PR China.

**Keywords:** yeast cell wall particle, PTOA, gene therapy, oral drug delivery, macrophage

## Abstract

Post-traumatic osteoarthritis (PTOA) is an acute injury-induced joint inflammation followed by a gradual degradation of articular cartilage. However, there is no FDA-approved Disease-Modifying Osteoarthritis Drug currently. Although gene therapy with microRNA can improve PTOA progression, there is no effective gene delivery vehicle for orally deliver therapeutics due to the harsh environment of the gastrointestinal tract. In this study, we investigated the effect of yeast cell wall particle (YCWP) mediated nanotube-RNA delivery system on PTOA therapy via oral route.

**Methods:** Nontoxic and degradable AAT and miRNA365 antagomir was self-assembled into miR365 antagomir/AAT (NPs). Then NPs-YCWP oral drug delivery system was constructed by using NPs and non-pathogenic YCWP which can be specifically recognized by macrophages. Moreover, surgical destabilization of the medial meniscus induced PTOA mice model was established to evaluate the therapeutic effect of NPs-YCWP via oral route.

**Results:** Compared with control group, NPs showed higher gene inhibition efficiency both in chondrogenic cell line and primary chondrocytes *in vitro*. Treatment of macrophages with fluorescently labeled NPs-YCWP, the results showed that NPs-YCWP was successfully engulfed by macrophages and participated in the regulation of gene expression *in vitro*. Under the protection of YCWP, miR365 antagomir/AAT passes through the gastrointestinal tract without degradation after oral administration. After NPs-YCWP therapy, the results of histological staining, gene and protein expression showed that miR365 antagomir/NPs-YCWP improved the symptom of PTOA.

**Conclusion:** Here, we constructed a biodegradable drug delivery system based on non-pathogenic YCWP and nanotubes, which can be used for PTOA therapy via the oral route. It suggests a new gene therapy strategy with YCWP mediated oral nano drug delivery system may serve as a platform for joint degeneration treatment.

## Introduction

Post-traumatic osteoarthritis (PTOA) is characterized by an acute injury-induced joint inflammation followed by a gradual degradation of articular cartilage 1, 2. Almost fifteen percent of the global population over 60 years suffers from PTOA 3 and whose further development will lead to disability. Anterior cruciate ligament meniscal surgery may restore joint function but does not affect PTOA pathogenesis 4. Current treatments are restricted to behavioral interventions and ultimately joint replacement surgery. There is no FDA-approved Disease-Modifying Osteoarthritis Drug for prevention and treatment of PTOA 5.

Previous studies showed miR365 was up-regulated in OA chondrocytes 6 and OA cartilage from primary and traumatic OA patients 7. Therefore, down-regulation the expression of miR365 by RNA interference method with miR365 inhibitor may be a potent therapeutic method for the treatment of OA, because gene therapy has become an attractive method for OA therapy 8-14 and diseases treament 15-17. How to effectively and safely deliver gene drugs into tissues has become a major obstacle to the use of this method.

Janus base nanotubes are new class of biomimetic supermolecule nanomaterial which formed from self-assembled of synthetic DNA base analogues (guanine-cytosine motif) 18. Previous studies have proved that nanotubes have unique chemical and physical properties 19 and can be used to transport small molecule RNA to cells and tissues with low toxicity, excellent biocompatibility and biodegradability 20. In order to widely apply this material, the way of administration route is crucial important. The oral route of drug administration is the most preferred due to its convenience, low cost, and high patient compliance. Compared with parenteral administration, oral route typically causes neither tissue damage nor pain and requires less patient supervision and decreased cost of care 21.

Despite the advantages of oral administration, oral uptake of many bio-therapeutics are limited by various physiological barriers such as the harsh gastro-intestinal environment, enzymatic degradation and oral tolerance which impede the clinical application of oral delivery systems 22. Different from bacteria, food grade non-pathogenic* Saccharomyces cerevisiae* has been demonstrated to avoid digestion in the stomach and small intestine 23 for gene delivery and been widely used in wine-making 24. The reason for interest in *S.cerevisiae* as a vaccine vehicle is its lack of toxicity and safety in humans 25. The other advantage of using *S.cerevisiae* as an oral delivery vehicle is its property of targeting antigen presenting cells (APCs) including macrophages (Mφ) and dendritic cells in the gastrointestinal tract. And beta-glucans are carbohydrate polymers on the yeast cell wall 26 which could be recognized by glucan receptor on the surface of macrophages 27. Recently, yeast microcapsule has been shown to be resistant to digestion in the gastrointestinal tract and be used for nanomaterials, gene and protein delivery via oral route 23, 28-31. It has been reported that drugs mediated by yeast microcapsules can be used for atherosclerosis therapy via oral route 30. Thus, these characteristics make yeast microcapsule a preferred delivery vehicle for disease treatment via oral route.

**Scheme 1:**
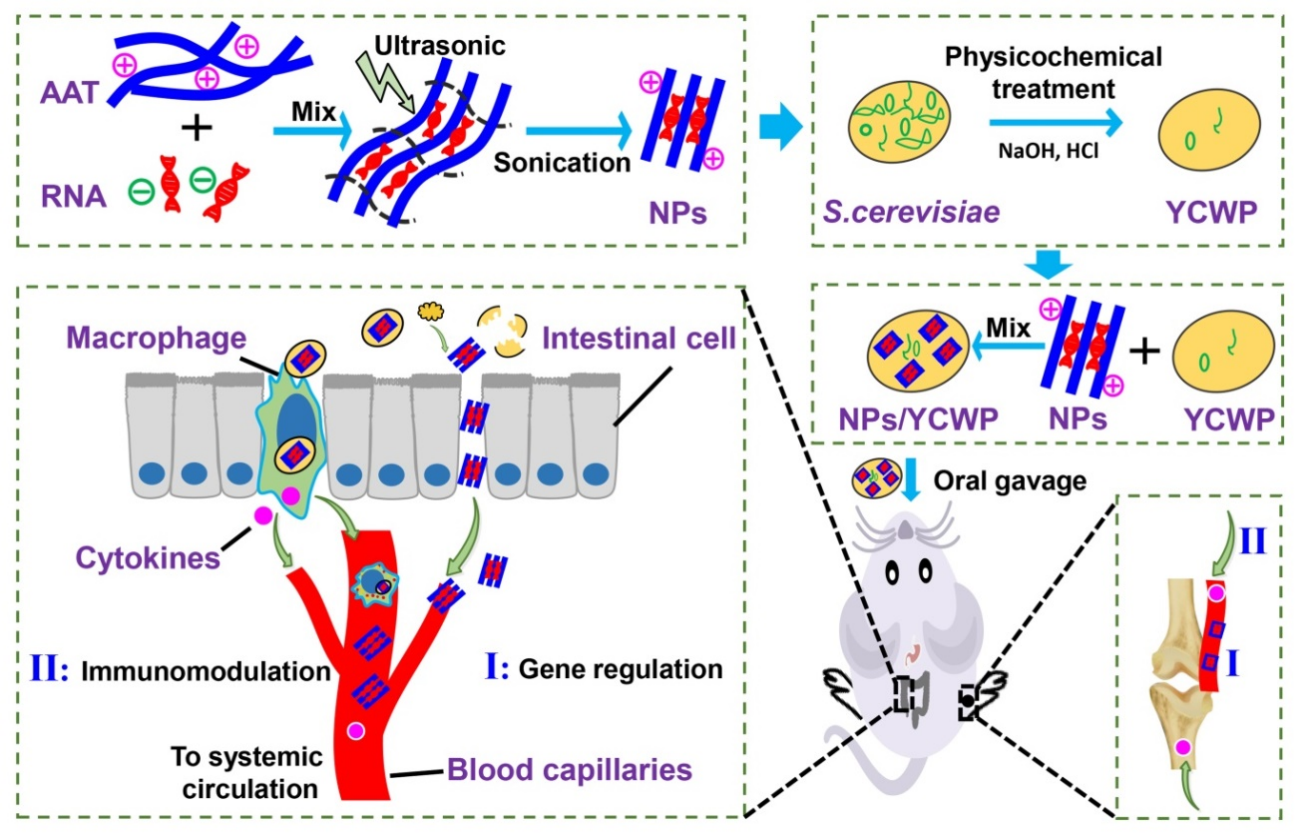
Schematically depicts the synthesis of NPs-YCWP and the application of miR365 antagomir/NPs-YCWP in PTOA therapy.

In this study, we hypothesized that by oral delivery miR365 antagomir via nanotubes AAT in yeast cell wall particle, we can inhibit miR365 content to treat PTOA. The aim of the present study was to develop a novel nano drug delivery system that can be used in the oral route to treat osteoarthritis under the assistance of yeast cell wall particle. The principle of this study is diagrammatically shown in Scheme 1.

## Results

### Functional detection of miR365 antagomir/NPs in vitro

**Figure 1:**
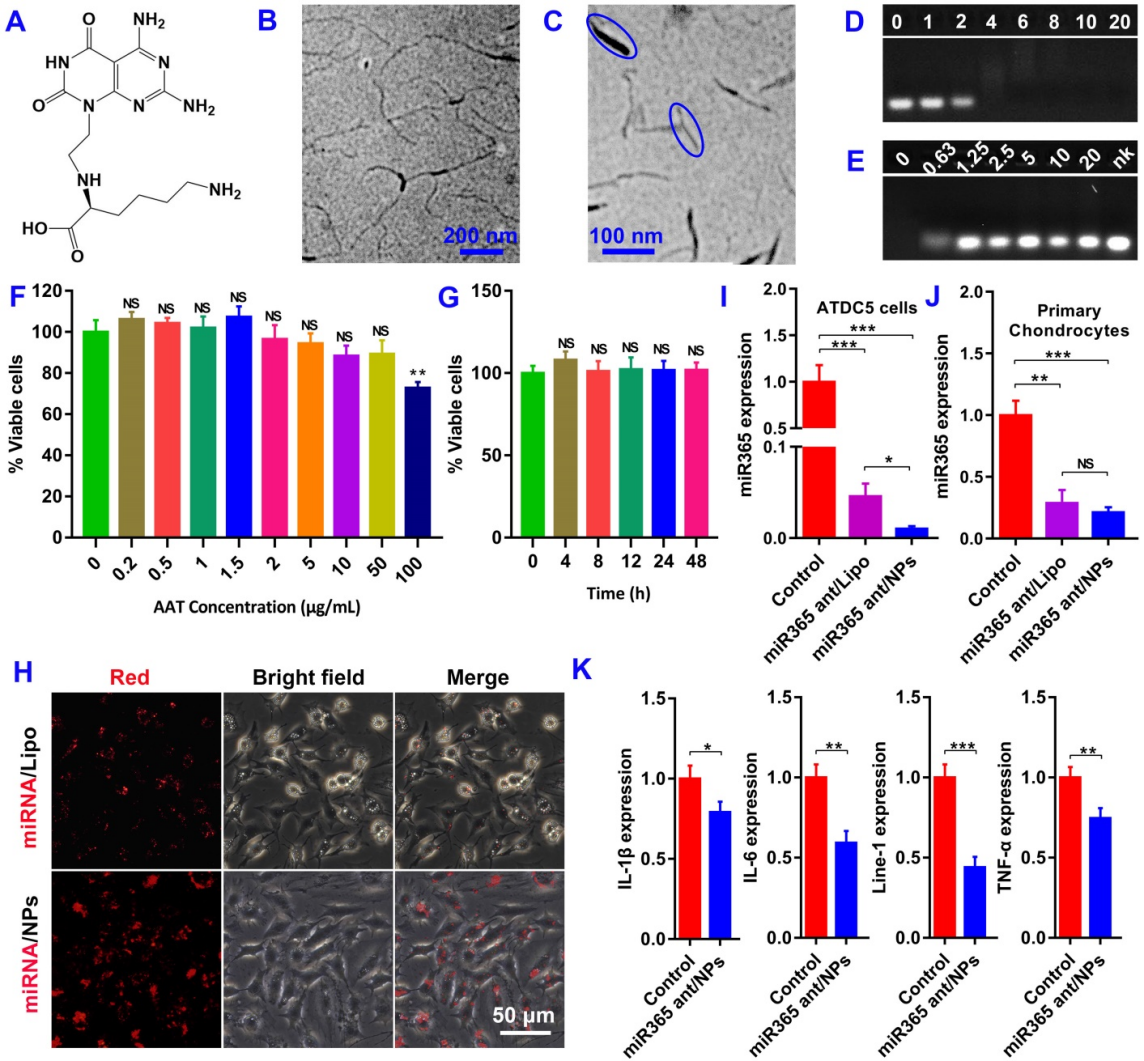
** Characterization of AAT and miR365 antagomir/NPs. (A)** The molecula structure of AAT monomer. Transmission electron microscopes (TEM) scan of nanotubes AAT **(B)** and miR365 antagomir/NPs **(C)**. **(D)** Agarose gel electrophoresis results of miR365 antagomir/NPs complexes at various v/v ratios (0, 1, 2, 4, 6, 8, 10 and 20 µL, 1 mg/mL). **(E)** Evaluate the release and stability of miR365 antagomir/NPs with different mass heparin sodium salt (0, 0.63, 1.25, 2.5, 5, 10, 20 mg/mL and nk-naked miRNA control). **(F)** Cytotoxicity of AAT at different concentrations on ATDC5 cells (All data was compared with AAT at 0 µg/mL concentration). **(G)** Cytotoxicity of AAT (10 µg/mL) on ATDC5 cells at different time points (All data was compared with cell treatment at 0 h). **(H)** Transfection efficiency of miRNA/AAT in ATDC5 cells. The Cy3 labeled negative control miRNA was used to detect AAT transfection efficiency. Compared with commercial transfection reagent Lipo2000, AAT (5 µg/mL) showed higher transfection efficiency in ATDC5 cells 12 h after transfection. Comparison of miRNA365 antagomir transfection efficiency between AAT and Lipo2000 in ATDC5 cells **(I)** and primary chondrocytes **(J)**. **(K)** The expression of IL-1β, IL-6, line-1 and TNF-α in ATDC5 cells after 24 h treatment with miR365 antagomir/NPs. Data were expressed as mean ± SD. NS (no significance). *P < 0.05, **P < 0.01, ***P < 0.001 (n=3).

The molecula structure of nanotubes AAT monomer was shown in Figure 1A. Transmission electron microscope (TEM) scan of nanotubes AAT and miR365 antagomir/NPs were shown in Figure 1B-C. The miR365 binding and complexes stability evaluation of AAT at different v/v ratios were carried out by agarose gel electrophoresis. AAT could completely bind miRNA at the v/v ratio of 6 (Figure 1D and S1A), suggesting the good gene binding performance. The miR365 antagomir/NPs which were self-assembled by miR365 antagomir and AAT nanotubes showed 101.2 ± 11.5 nm size (measured by TEM). To detect the release of miR365 antagomir from miR365 antagomir/NPs nanocomplex, the heparin sodium salt as a polyanion to evaluate the controlled release of miRNA/NPs. The miR365 antagomir could be released from miRNA/NPs when the heparin sodium salt concentration was higher than 0.63 mg/mL (Figure 1E). When the concentration of heparin sodium was 1.25 mg/mL, miRNA can be almost completely released from miRNA/NPs (Figure S1B). The chondrogenic cell line ATDC5 cells were employed to analyze the cytotoxicity of AAT. Our results showed that AAT presented no cytotoxicity at the dose below 50 µg/mL (Figure 1F). And AAT (10 μg/mL) showed no cytotoxicity on ATDC5 cells at 24 h (Figure 1G). The Cy3 fluorescence-labeled negative control miRNA was used to detect whether AAT has the ability to deliver gene into cells. Compared with commercial transfection reagent lipofectamine 2000 (Lipo2000), miRNA/NPs showed higher transfection efficiency than miRNA/Lipo2000 in ATDC5 cells (Figure 1H-I). However, there was no significant difference in transfection efficiency between miRNA/NPs and miRNA/Lipo2000 group in the primary chondrocytes (Figure 1J). Compared with control, miR365 antagomir/NPs can not only inhibit miR365 expression, but also regulate IL1β, line-1 and TNF-α expression (Figure 1K). These results indicate that AAT is not only non-toxic, but also has good gene delivery ability.

### Preparation of fluorescently labeled YCWP and NPs-gYCWP structure detection

**Figure 2:**
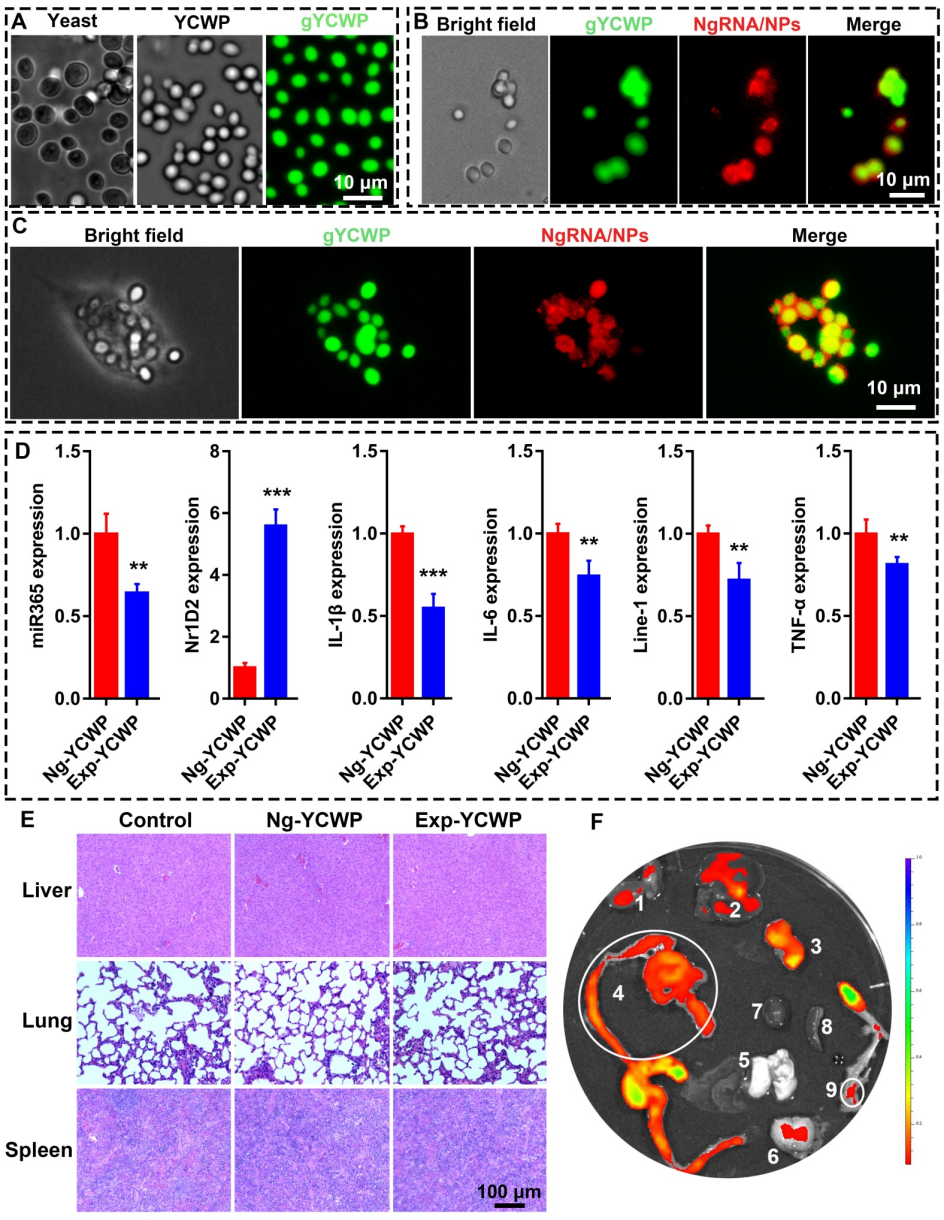
** Construct NPs-YCWP and its functional detection.** (**A**) The image of yeast, YCWP, green labeled gYCWP was collected by microscope. (**B**) Imaging of NgRNA/NPs-gYCWP under fluorescence microscope. (**C**) NgRNA/NPs-gYCWP (self-assembled with red labeled NgRNA/NPs and green labeled gYCWP) was engulfed by the LPS induced RAW264.7 macrophages. (**D**) Negative control NgmiRNA/NPs-YCWP (Ng-YCWP) or miR365 antagomir/NPs-YCWP (Exp-YCWP) containing 50 nM miRNA were added into LPS induced RAW264.7 macrophages for 24 h. Then RNA was extracted for miR365, IL-1β, IL-6, Nr1D2, line-1 and TNF-α quantification (n=3).** (E)** Evaluated the *in vivo* tissue toxicity of miR365 antagomir/NPs-YCWP (n=4). **(F)** The fluorescence signals of organs were detected by PerkinElmer (1. kidney, 2. liver, 3. stomach, 4. small intestine, 5. lung, 6. brain, 7. heart, 8. spleen and 9. knee joint) (n=3). Data were expressed as mean ± SD. **P < 0.01, ***P < 0.001 versus control group.

*S. cerevisiae* was used for yeast cell wall particle (YCWP) preparation by alkaline-and-acid extraction and then treatment with isopropanol and acetone. The eluate produced during the chemical treatment was shown in Figure S2. Then, 5-(4,6-dichlorotriazinyl) aminofluorescein (Thermo; 1 mg/mL in DMSO) was used to label YCWP to get green gYCWP (Figure 2A and S3A). NgRNA/NPs-gYCWP was constructed with green fluorescently labeled gYCWP and Cy3 red labeled NgRNA/NPs by the method described in Methods. The only difference between NgRNA/NPs-gYCWP (Figure S3B) and NgRNA-AAT-gYCWP (Figure S3C) was that we did not pre-assemble NgRNA and AAT into NPs during NgRNA-AAT-gYCWP construction process. And the TEM scan of YCWP and NPs-YCWP were shown in Figure S4. These results showed us that only when RNA and AAT were prepared into NPs in advance can they be delivered into YCWP. It means that unassembled AAT cannot directly deliver RNA into YCWP. This may be due to the fact that the size of AAT is much larger than NPs, which makes AAT unable to transfer RNA to YCWP through synergy.

### NPs-YCWP be engulfed by macrophages and participated in the regulation of gene expression

Before functional detection of NPs-YCWP drug delivery system, we need to verify whether our NPs-YCWP system can be successfully recognized and engulfed by macrophages. Firstly, we used red labeled NgRNA/NPs and green labeled gYCWP to assemble NgRNA/NPs-gYCWP (Figure 2B). NgRNA/NPs-gYCWP was added in the LPS induced RAW264.7 macrophages culture medium, and incubated at 37 °C under 5% CO_2_ for 4 h. We observed that NgRNA/NPs-gYCWP was engulfed by macrophages (Figure 2C). This means that NPs-YCWP system can be successfully recognized and engulfed by macrophages.

Then 10^5^/6-well negative control NgmiRNA NPs-YCWP (Ng-YCWP) or miR365 antagomir NPs-YCWP (Exp-YCWP) containing 50 nM miRNA were added into LPS induced RAW264.7 macrophages culture medium, and incubated at 37 °C under 5% CO_2_ for 24 h. Cells were harvested for RNA isolation. The gene expression of miR365, IL-1β, IL-6, Nr1D2, Line-1 and TNF-α was quantified by RT-qPCR (Figure 2D). Compared with the control group Ng-YCWP (NgmiRNA/NPs-YCWP), the experimental group Exp-YCWP (miR365 antagomir/NPs-YCWP) could knock-down miRNA365 expression* in vitro*. And the expression of IL-1β, IL-6, Line-1 and TNF-α were down regulated but Nr1D2 was up regulated when down regulation of miR365* in vitro*.

### Non-toxic NPs-YCWP has the ability to deliver gene drugs orally

Before analysing the *in vivo* PTOA therapy capacity, we evaluated the *in vivo* tissue toxicity of miR365 antagomir/NPs-YCWP. Mice were orally administrated with miR365 antagomir/NPs-YCWP or NgmiRNA/NPs-YCWP (10^6^/YCWP with 100 pmol miR365 antagomir or NgmiRNA) every two days for 50 days. Then liver, lung and spleen of mice were collected for hematoxylin-eosin (H&E) staining. Compared with the control (treatment with PBS), there was no significant difference in tissue integrity and cell structure in NgmiRNA/NPs-YCWP (Ng-YCWP) and miR365antagomir/NPs-YCWP (Exp-YCWP) group. It means that miR365 antagomir/NPs-YCWP has no toxic effect on tissues* in vivo* (Figure 2E). According to NPs-YCWP construction method, we used Cy5 fluorescent labeled RNA to construct Cy5RNA/NPs-YCWP. Mice were orally administrated with Cy5RNA/NPs-YCWP (10^6^ YCWP with 1 nmol Cy5RNA) every 10 h for 3 times. The fluorescence signals of organs such as heart, liver, spleen, lung and kidney, brain, stomach and leg were detected by PerkinElmer (PerkinElmer IVIS Lumina XR Series III). Fluorescence signals could be detected in the small intestine, knee joint, kidney and liver (Figure 2F). *Ex vivo* semi-quantitative anslysis of fluorescence biodistribution was showed in Figure S5. This showed that NPs-YCWP can deliver RNA drugs to knee joint via oral administration.

### NPs be protected by NPs-YCWP in simulated gastrointestinal fluid *in vitro*

**Figure 3:**
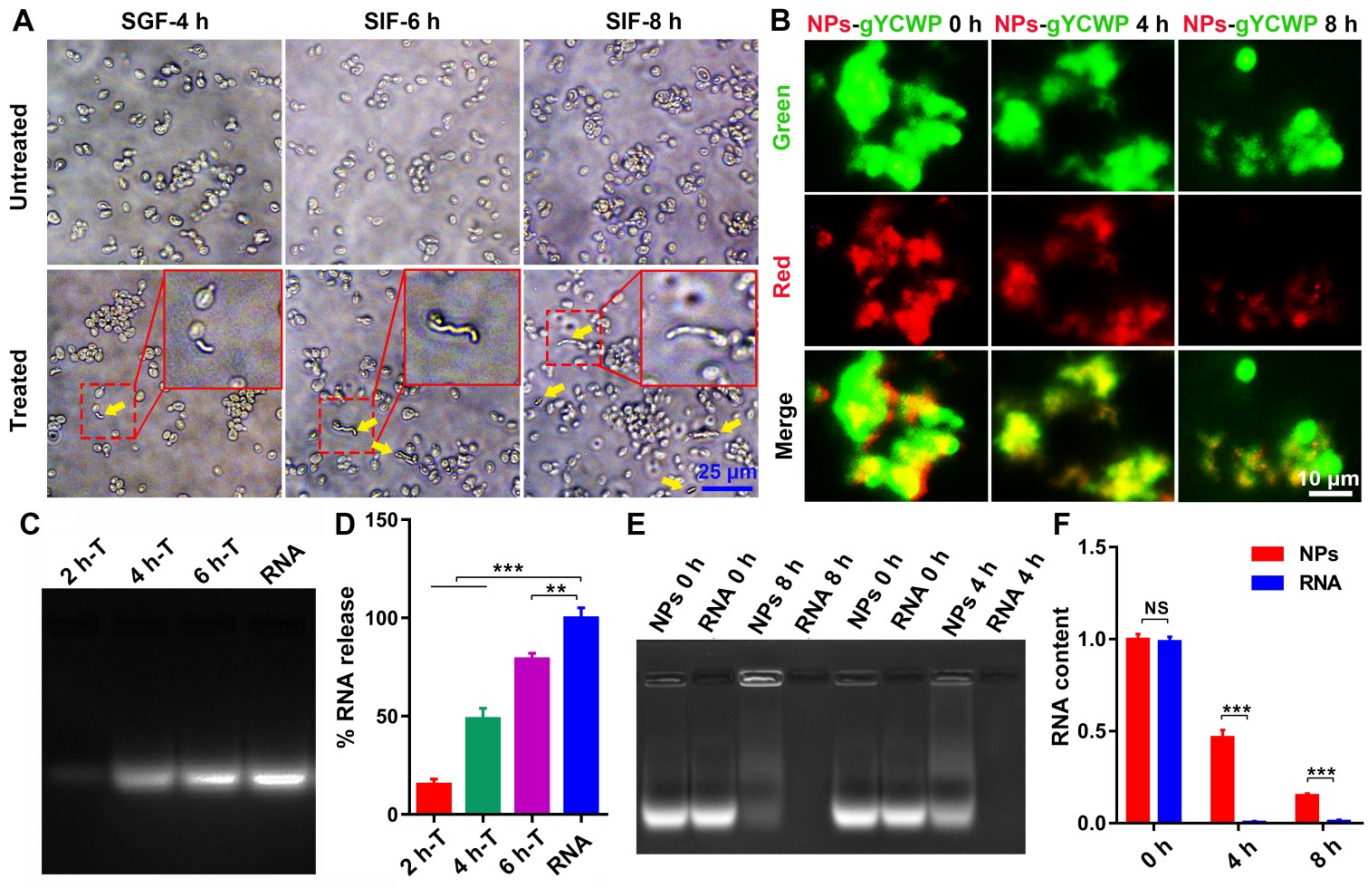
** NPs were protected by NPs-YCWP in simulated gastrointestinal fluid *in vitro.* (A)** Simulated gastric fluid (SGF) and simulated intestinal fluid (SIF) were used to assess the stability of YCWP. 10^5^ YCWP was added into 500 µL of SGF and incubated for 4 h at 37 °C. Then resuspended the YCWP with 500 µL SIF and incubated for 6-8 h at 37 °C. Yellow arrows indicated the lysed yeast cell wall. **(B)** Detect the protective effect of NPs-YCWP on NPs in SGF. NPs-gYCWP with red labeled negative control RNA was used to detect the protective effect of NPs-YCWP on NPs (Cy3RNA/AAT) at a pH 1.2 of SGF. 10^5^ YCWP was added into 500 µL of SGF and incubated in the dark for 4-8 h at 37 °C. **(C-D)** NPs can be released from NPs-YCWP in SIF. NPs-YCWP was incubated with 50 µL SIF for 2-6 h at 37 °C. Next, equal volume of heparin sodium salt (5 mg/mL) was added and incubated for 1 h at 37 °C. Then RNA intensity was measured using an agarose gel retardation assay (2 h-T, 4 h-T and 6 h-T represent NPs-YCWP was treated with SIF for 2 h, 4 h and 6 h respectively). (D) Quantification of % RNA content. **(E-F)** Evaluate the degradation cycle of NPs in SIF by measuring RNA content. NPs were incubated in SIF for 4 or 8 h at 37 °C (naked RNA as control). Then added equal volume of heparin sodium salt (5 mg/mL) and incubated for 1 h at 37 °C. (E) The RNA intensity was measured using an agarose gel retardation assay (NPs 0 h or RNA 0 h represents NPs or naked RNA without SIF treatment; NPs 4 h or RNA 4 h represents NPs or RNA was treated by SIF for 4 h). (F) Quantification of RNA content. Data were expressed as mean ± SD. NS (no significance). **P < 0.01, ***P < 0.001 versus control group (n=3).

Simulated gastric fluid (SGF) and simulated intestinal fluid (SIF) 32 were used to evaluate the mechanism of YCWP-NPs. Our results showed that most of YCWP can effectively resist the corrosion of SGF. After further treatment by SIF, YCWP began to be degraded gradually (Figure 3A). And as time went on, the phenomenon of degradation was intensified. This indicated that YCWP can resist the corrosion of gastrointestinal fluid to a certain extent, but it cannot completely resistant to degradation. Then we treated the fluorescent labeled Cy3RNA/NPs-gYCWP (red NPs and green gYCWP) with acid solution (pH 1.2) to detect the protective effect of YCWP on NPs. After 8 h of treatment with SGF, NPs can still be observed in the gYCWP (Figure 3B).

With 8 h of treatment in SIF, although some of the fully degraded YCWP were observed, many of the cell structures of YCWP still existed. However, compared with the untreated group, the volume of YCWP with intact structure decreased significantly. We speculated that NPs have been released into the simulation liquid from the YCWP. To prove this, we treated NPs-YCWP with SIF and tested whether NPs was released from YCWP or not. If NPs were released from YCWP, they can be degraded by the added heparin sodium salt, and then RNA was separated from NPs. We found that the 6 h treatment group had a large number of NPs released compared to the 2 h treatment group (Figure 3C-D). Then we tested the degradation cycle of released NPs in SIF (pH 6.8). Compared with naked RNA, NPs can effectively protect RNA from degradation by SIF (Figure 3E-F). After 4 h of treatment, about 47% of RNA was detected (Figure 3F). This suggests that after NPs-YCWP carries NPs to the small intestine, the released NPs from YCWP still has a half-life cycle of 4 h to enter the humoral circulation and participate in gene regulation.

### MiR365 antagomir/NPs-YCWP inhibits the inflammatory response of PTOA mice

**Figure 4:**
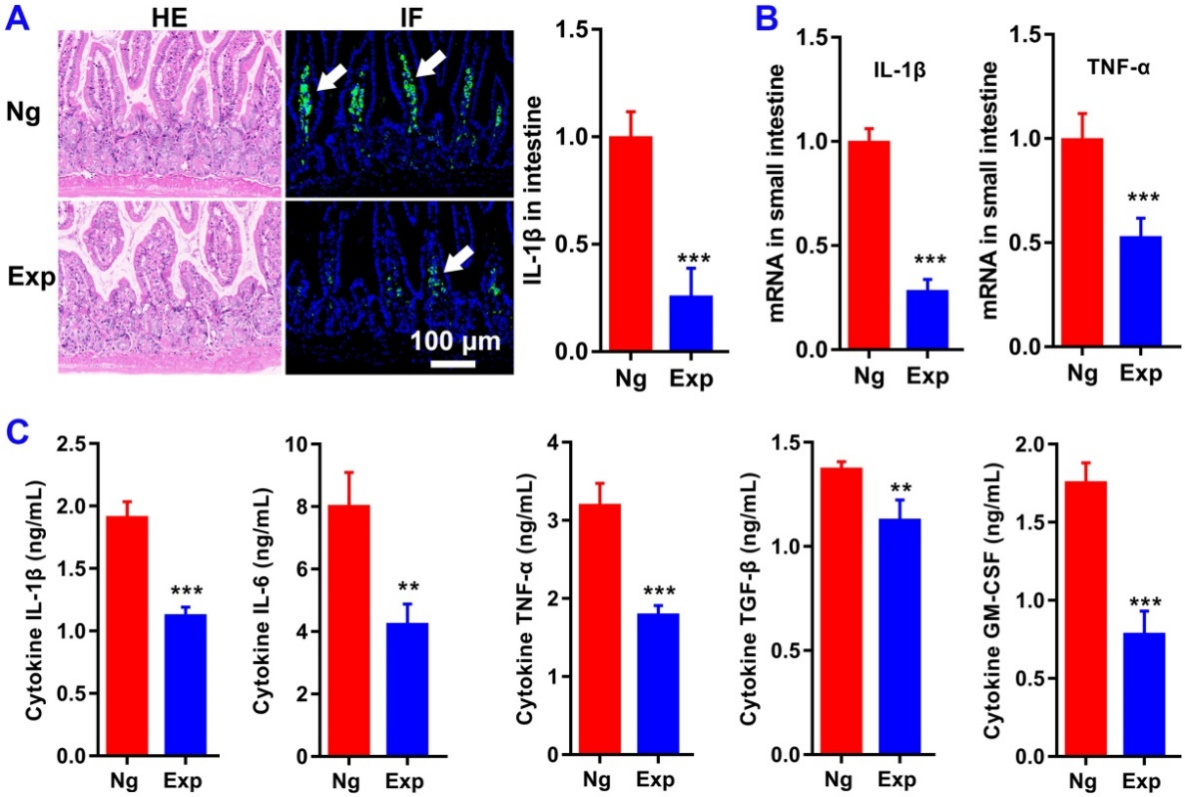
** MiR365 antagomir/NPs-YCWP inhibits the inflammatory response of PTOA mice.** After oral administration of miR365 antagomir/NPs-YCWP (Exp, 10^6^/YCWP with 100 pmol miR365 antagomir) or NgmiRNA/NPS-YCWP (Ng, 10^6^/YCWP with 100 pmol negative control antagomir) every two days for 50 days, serum and small intestine were collected for ELISA and histological staining. (**A**) HE and IL-1β immunofluorescence staining of small intestine. (**B**) Inflammatory gene IL-1β and TNF-α in small intestine were inhibited at mRNA level. (**C**) Cytokines IL-1β, IL-6, TNF-α, TGF-β and GM-CSF expression in serum. Data was expressed as mean ± SD. **P < 0.01, ***P < 0.001 versus control group (n=8).

To evaluate the function of miR365 antagomir/NPs-YCWP on the treatment of OA* in vivo*, a PTOA murine model via surgical destabilization of the medial meniscus (DMM) was used. After oral administration of miR365 antagomir/NPs-YCWP (10^6^/YCWP with 100 pmol miR365antagomir) every two days for 50 days, serum and small intestine were collected for enzyme linked immunosorbent assay (ELISA) and histological staining. Oral treatment with miR365 antagomir/NPs-YCWP knocked down inflammatory protein IL-1β expression in the small intestine (Figure 4A). And qPCR results also showed that inflammatory gene IL-1β and TNF-α in the small intestine were inhibited at mRNA level (Figure 4B). To determine whether cytokine secretion was affected by miR365 antagomir/NPs-YCWP, here we quantify the cytokines expression in serum. And the ELISA results showed that inflammatory cytokine IL-1β, IL-6 and TNF-α expression (**P < 0.01, ***P < 0.001) in serum was down regulated after inhibition of miR365 expression (Figure 4C).

### Oral administration of miR365 antagomir/NPs-YCWP improve the symptoms of PTOA

**Figure 5:**
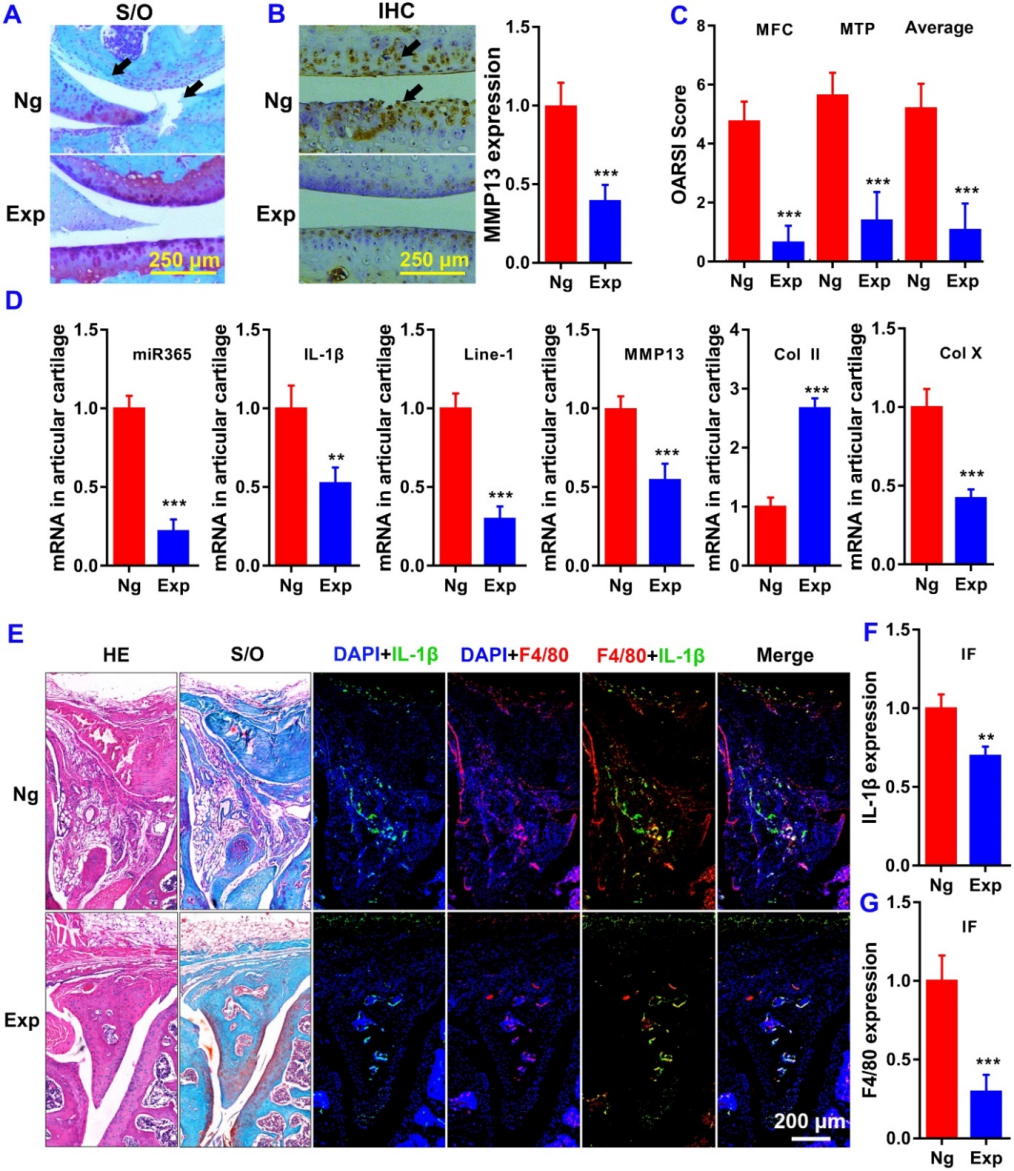
** MiR365 antagomir/NPs-YCWP improved the symptoms of PTOA.** (**A**) Safranin O/Fast Green stainning (Black arrows in S/O staining indicated the cartilage lesion sites). (**B**) Immunohistochemistry staining of MMP13 (Black arrows in the IHC staining indicated high expression of MMP13). Protein MMP13 expression in articular cartilage was quantified. (**C**) The OA score of medial femoral condyle (MFC), the medial tibial plateau (MTP) and average of the MFC and MTP values. (**D**) OA realted gene expression in articular cartilage. (**E**) Histological staining of the meniscus. OA related gene (**F**) IL-1β (green) and macrophage specific marker (**G**) F4/80 (red) expression in meniscus of PTOA mice. Data was expressed as mean ± SD. **P < 0.01, ***P < 0.001 versus control group. This is representative data of four repeats (n=4).

We performed histological analysis using safranin O/fast green (S/O) for knee joint staining. Histological examination showed that treatment with miR365 antagomir/NPs-YCWP (Exp) attenuated cartilage lesion formation. Whereas stronger Safranin O staining with less loss of the superficial layer was observed in the Exp group who had stronger proteoglycan staining and more intact surface than the NgmiRNA/NPs-YCWP group (Ng) (Figure 5A). And immunohistochemistry result revealed that OA related protein MMP13 staining in the Ng group was stronger than that in the Exp group (Figure 5B). Highly expressed MMP13 increases the risk of cartilage lesion formation in the Ng group. The osteoarthritic damage score was assessed individually by three professional observers using OARSI criteria. The score of medial femoral condyle (MFC) and the medial tibial plateau (MTP) in the Ng group is much higher than that in the Exp group (Figure 5C).

Oral delivery of miR365 antagomir/NPs-YCWP significantly elevated anabolic cartilage matrix marker col2a1 (Col II) level while suppressed hypertrophic marker col10a1 (Col X) level in cartilage joint (Figure 5D). And qPCR results also showed that miR365, IL-1β, Line-1 and MMP13 in articular cartilage were inhibited at mRNA level (Figure 5D). We also used macrophages specific expression tag F4/80 and OA-related gene IL-1β to study the function of macrophages in OA articular cartilage. Compared with the Exp group, more F4/80 and IL-1β expression were detected both in meniscus (Figure 5E-G), distal femur and proximal tibia (Figure S6) than those in the Ng group. The results indicated that the inflammatory reaction in Ng group knee joints was more serious than that in the Exp group. Those results suggest that the degradation of articular cartilage was alleviated by YCWP which contained miR365 antagomir/NPs via oral administration method.

### MiR365 antagormir/NPs-YCWP regulated gene expression *in vivo*

**Figure 6:**
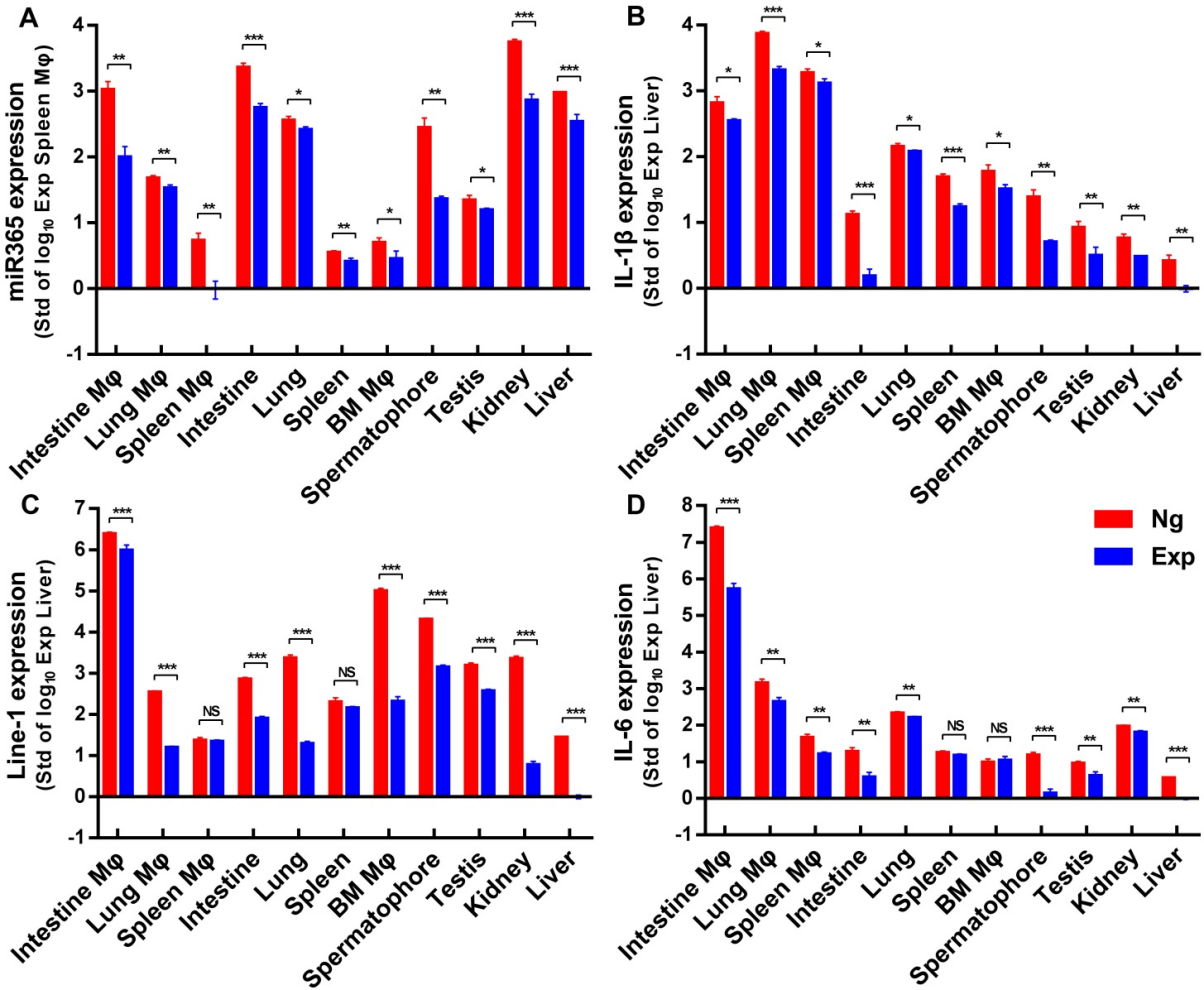
** MiR365 antagomir/NPs-YCWP regulated PTOA related functional genes expression* in vivo*.** After oral administration of miR365 antagomir/NPs-YCWP (10^6^/YCWP with 100 pmol miR365 antagomir) for 50 days, small intestine, lung, spleen, bone marrow, spermatophore, testis, kidney and liver were collected for gene expression quantification. CD11b MicroBeads were used for sorting macrophages (Mφ) from small intestine, lung, spleen and bone marrow. MiR365 **(A)**, IL-1β **(B)**, Line-1** (C)** and IL-6 **(D)** expression from those macrophages and tissue cells were quantified by RT-qPCR. For each gene expression detect, we logarithmized the data and compared them to the smallest expressed tissue cells. Data were expressed as mean ± SD. NS (no significance). *P < 0.05, **P < 0.01, ***P < 0.001 versus control group (n=4).

After oral administration of miR365 antagomir/NPs-YCWP for 50 days, some organs or tissues (lung, spleen, small intestine, liver, kidney, testis, spermatophore and bone marrow) were collected to detect genes expression. The gene expression of miR365, IL-1β, line-1 and IL-6 (Nr1D2, TNF-α and Arg-1 expression were shown in Figure S7) in those tissue cells (Figure 6) were quantified by RT-qPCR. Compared with the Ng group (NgmiRNA/NPs-YCWP), the expression of miR365, IL-1β, line-1 and IL-6 in the Exp group (miR365 antagomir/NPs-YCWP) were significantly down-regulated in most of the tissue cells. The loss of miRNA functional phenotypes induced by miRNA antagomir has been widely used in many diseases studies 33-35. This antagonistic effect is different from gene targeting 36, 37 or gene editing 38, 39, which permanently silences a gene expression. This short-term blocking of miR365 content can effectively control the expression of IL-1β, IL-6 and MMP13 which accelerate the injury of articular cartilage. MiR365 antagomir competitively bind to miR365 and specifically inhibit miR365 content. Then low expression of IL-1β, IL-6 and MMP13 promotes articular cartilage repair.

REV-ERBs (Nr1D1 and Nr1D2) are transcription repressors and circadian regulators which involved in the NF-kB signaling pathway 40. Previous studies showed that circadian clock gene such as Nr1d1 and BMAL1 (brain and muscle arnt-like 1) were involved in osteoarthritis 41, 42. REV-ERBs repress the expression of target genes, including MMP9 in macrophages 40. In addition, the synthetic REV-ERB agonist SR9009 reduces the polarization of bone marrow-derived mouse macrophages (BMMs) to pro-inflammatory M1 macrophages while increasing the polarization of BMMs to anti-inflammatory M2 macrophages 43. In this study, we found that down regulation of miR365 expression up-regulated the expression of Nr1D2 *in vitro.* Compared with the control group, up-regulation of Nr1D2 in intestine macrophages, bone marrow macroohages and lung macrophages, but down-regulation in tissue cells was observed in the Exp group. This may be because tissue cells (not antigen presenting cells) are unable to uptake NPs-YCWP directly like macrophages.

Retrotransposons are genetic elements that move through RNA intermediate in the process of retrotransposotion. Previous studies indicate that Line-1 plays a role in the activation of rheumatoid arthritis 44. Recently, it has been shown that Line-1 is a late cell senescence marker, which plays an important role in triggering sterile inflammation during aging 45. Our *in vitro* result showed that the expression of Line-1 in the experimental group was inhibited when miR365 down regulated. In addition to spleen and spleen macrophages, the expression of line-1 was significantly decreased in the other 9 cells* in vivo*. This is the first time to show that Line-1 is related to OA and could be regulated by using miR365 antagomir/NPs-YCWP *in vivo*.

Macrophages (Mφ) can be divided into two types according to the activation status: classically activated M1 and alternatively activated M2 46, 47. Type M1 can secrete proinflammatory cytokines and participate in antigen presentation activity and immune response 47. Type M2 participate in immunoregulation by secreting anti-inflammatory cytokines 46. Evidence suggests that yeast can be engulfed by gut-associated lymphatic tissue macrophages via oral route. Here we detected M1 correlative tag TNF-α and M2 correlative tag arginase-1 (Arg-1) and found that both of the two genes were down regulated in the Mφ from intestine, lung, spleen and bone marrow after miR365 antagomir/NPs-YCWP therapy. The down-regulation of TNF-α in the experimental group (Exp) indicated that the inflammatory reaction in the Ng group was more serious than that in the Exp group. Noteworthy, the Arg-1 expression in the Exp group was also significantly lower than that in the control group. We speculate that this may be because the PTOA symptoms in the Exp group mice are basically treated, and the body no longer needs too many M2 cells to anti-inflammatory response, so as to maintain the dynamic balance of the immune system 48-50.

## Discussion

PTOA has been characterized by the degradation of articular cartilage and joint inflammation and whose further development will lead to disability. Previous studies showed that miR365 was up-regulated in OA chondrocytes 6 and OA cartilage from primary and traumatic OA patients. And the overexpression of miR365 in chondrocytes could increase the expression of MMP13 and collagen X (Col X), which can aggravate the occurrence of OA 7. Therefore, we hypothesize that down-regulation of miR365 expression may be a potent therapeutic method for OA treatment, as gene therapy strategies that target on mRNA or miRNA were widely used in osteoarthritis or cartilage repair 8-10, 13, 14.

In this study, our main purpose was to use nanotube-mediated miR365 antagomir to suppress miR365, which is highly expressed at PTOA knee joint, by gene therapy. As we know, the oral route of drug administration is the most preferred due to its convenience, low cost and high patient compliance. Compared with parenteral administration, oral route of drug administration is the most preferred due to it causes neither tissue damage nor pain and requires less patient supervision and decreased cost of care 21. Although this strategy is very practical, oral administration inevitably needs to overcome various physiological barriers such as the harsh gastro-intestinal environment, enzymatic degradation. To solve this problem, we developed a novel oral gene therapy strategy using yeast cell wall particle (YCWP) as a delivery vehicle. There are three major reasons of choosing YCWP as oral delivery vehicle. First, often called brewer's/baker's yeast, *S.cerevisiae* is safe and widely used for oral consumption in food, wine, and beer. Second, *S.cerevisiae* has been shown to be an effective vehicle for gene delivery to the gut. Different from bacteria, food grade non-pathogenic *Saccharomyces cerevisiae* has been demonstrated to avoid digestion in the stomach and small intestine 23. Third, beta-glucans are carbohydrate polymers on the yeast cell wall 26 which could be specifically recognized by glucan receptor on the surface of macrophages 27. Evidence suggests that yeast was engulfed by gut-associated lymphatic tissue macrophages via oral route. And macrophages in the gut-associated lymphatic tissue may traffic away from the gut and infiltrate other reticuloendothelial and mononuclear phagocyte system tissues 30, 31, 51, 52. It has been reported that drugs mediated by yeast microcapsules can be used for atherosclerosis therapy via oral route 30.

In addition to the previous evidence of “macrophages in the gut-associated lymphatic tissue may traffic away from the gut and infiltrate other reticuloendothelial and mononuclear phagocyte system tissues 30, 31, 51, 52”, we speculate that NPs-mediated gene regulation plays an important role in the treatment of PTOA in this study. To prove this hypothesis, simulated gastric fluid (SGF) and simulated intestinal fluid (SIF) 32 were used to evaluate the mechanism of YCWP-NPs. Our results showed that YCWP can resist the corrosion of gastrointestinal fluid to a certain extent, but it cannot completely resistant to degradation. Compared with the untreated group, the volume of YCWP with intact structure decreased significantly after SIF treatment. This is because NPs were released into the SIF from the YCWP. And the released NPs can effectively protect RNA from degradation by SIF. This suggests that after NPs-YCWP carries NPs to the small intestine, the released NPs from YCWP may enter the humoral circulation and participate in gene regulation.

IL-1β is one of the key genes involved in the pathogenesis of PTOA 53. It can promote the production of matrix metalloproteinases (MMPs), enhance collagen and proteoglycan breakdown and release in cartilage 54. IL-6 is an inflammatory cytokine closely related to cell injury and senescence 53. IL-6 increased MMPs mRNA levels and intra-articular injection of IL-6 lead to OA like cartilage lesion in mice 55. Our *in vitro* results have shown that when miR365 expression was inhibited, the expression of IL-1β, IL-6 and TNF-α was down-regulated both* in vitro* and *in vivo*. As genes are suppressed, the inflammatory proteins (or cytokines) translated by these genes were significantly reduced. In turn, the down regulated expression of inflammatory cytokines has a positive effect on the treatment of PTOA, which has a certain auxiliary therapeutic effect on PTOA.

Safranin O/Fast Green staining showed that less losses of the superficial layer was observed in the miR365 antagomir/NPs-YCWP treated PTOA mice. And MMP13 IHC staining results also confirmed this conclusion. Highly expressed MMP13 means cartilage degradation in the control group. We also used macrophages specific expression tag F4/80 and OA-related gene IL-1β to study the function of macrophages in PTOA articular cartilage. Compared with the Exp group, more F4/80 and IL-1β expression were detected in meniscus, distal femur and proximal tibia. This indicated that the inflammatory reaction in the joints of the Ng group was more serious than that in the Exp group, which meant the degradation of articular cartilage was alleviated by miR365 antagomir/NPs-YCWP via oral administration method.

There were several limitations in this study which could be done better in the future. First, a large number of studies have shown that yeast can be engulfed by gut-associated lymphatic tissue macrophages via the oral route. And macrophages in the gut-associated lymphatic tissue may traffic away from the gut and infiltrate other reticuloendothelial and mononuclear phagocyte system tissues 30, 31, 51, 52. Although we can proved that NPs was indeed transferred from the gut to the knee joint and knock down the gene expression in articular cartilage, there was no direct imaging evidence to support this conclusion that YCWP was engulfed by intestinal macrophages and transferred to the knee joint in this study. More information about whether macrophages might be trafficked from the gut to the synovium through vasculature in the synovium should be studied in the future. Second, more studies will be needed and measured about relationships between PTOA pathogenesis and circadian rhythm and retrotransposons genes as data related to just one gene in each family was presented. Third, the safety of this oral gene therapy needs to be verified by more comprehensive testing. Although loss of miRNA functional phenotypes induced by miRNA antagonists have been widely used in many diseases studies 33-35, it is worth further study by increasing the tissue specificity of the drug delivery system.

In conclusion, we created an efficient miRNA delivery system via orally administered yeast cell wall particle for PTOA treatment *in vivo* (in mice). This is the first study to demonstrate the feasibility of alleviating experimental PTOA by using oral administration of NPs-YCWP as gene therapy vehicle. Such oral gene therapy may have strong potentials for treatment of degenerative diseases in addition to PTOA as demonstrated here.

## Materials and Methods

### Study design

The objective of this study was to create a novel and efficient nanotubes based small RNA delivery system that could be used for post-traumatic osteoarthritis treatment via oral administration approach *in vivo*. Self-assembled RNA/NPs were constructed with positively charged nanotubes and negatively charged miR365 antagomir. *S. cerevisiae* was used for YCWP preparation by chemical treatment, which could protect RNA/NPs passing through the gastrointestinal environment after oral administration of RNA/NPs-YCWP via gavage. Post-traumatic osteoarthritis model mice were generated by surgical destabilization of the medial meniscus. Mice were randomly assigned to experimental or control group. All mice were treated according to the policy and regulations for care and use of laboratory animals. CD11b MicroBeads were used for sorting macrophages from different tissues. The therapeutic effects of this miR365 antagomir/NPs-YCWP nano-drug delivery system on PTOA mice were respectively evaluated by histological, mRNA, protein and serological levels.

### Gene binding and stability investigations of AAT nanotubes

Nanotubes AAT was synthesized according to the method described previously 18, 20. The gene binding ability of AAT was measured using an agarose gel retardation assay as our group described earlier 56. Briefly, here we fix the amount of miRNA (1 µL, 25 uM miRNA), and then use different doses of AAT (0, 1, 2, 4, 6, 8, 10 and 20 μg) to detect the optimal combination ratio of materials and genes. Heparin sodium salt as a polyanion was used to evaluate the release and stability of miRNA/AAT. Briefly, the complexes solution with v/v ratios of 5:1 was incubated with different mass heparin sodium salt (0, 0.63, 1.25, 2.5, 5, 10, 20 mg/mL) for 1 h at 37 °C and naked miRNA as control. The release of gene was detected using agarose gel assay as previously described. More detailed steps can be found in the supplementary materials.

### Preparation and characterization of miRNA/AAT

The miR365 antagomir/NPs nanomaterial was self-assembled with miR365 antagomir and AAT nanotubes. Briefly, add 2 µL of 25 µM miR365 antagomir (RiboBio, Guangzhou, China) into 10 µL RNase-free water and fully mixed. Then 10 µg nanotubes AAT be added into the mixture and sonicate at 100 amplitude for 150 s (Q700, Qsonica, USA). Positively charged nanotubes and negatively charged miR365 antagomir will form a sandwich structure of miR365 antagomir/NPs through electrostatic interaction. Briefly keep the miR365 antagomir nanopieces (miR365 antagomir/NPs) on ice before use. The particle size of miR365 antagomir/NPs was measured by transmission electron microscopy (TEM) (Nippon Tekno Co., Ltd). The sequence of miR365 antagomir was shown in Table S1.

### Cytotoxicity and gene transfection ability

Chondrogenic cell line ATDC5 cells were employed to investigate the cytotoxicity of AAT. Briefly, cells were seeded into 96-well plates at a density of 5000 cells/well and cultured in high glucose Dulbecco's modified Eagle's medium (DMEM, cat No.SH30022.01, HyClone, China) containing 10% FBS (cat No.10091148, Gibco, New Zealand) at 37 °C under a humidified atmosphere containing 5% CO_2_. After 24 h, the culture medium was replaced by DMEM with different concentrations of AAT (0, 0.2, 0.5, 1, 1.5, 2, 5, 10, 50 and 100 µg/mL). Subsequently, cell viability was investigated using CCK-8 (10 µL of CCK-8 was added into each well) assay by measuring the fluorescent intensity at an excitation/emission wavelength of 450/630 nm with Thermo Fisher Varioskan Flash (Thermo, Waltham, MA, USA) after cultured for 24 h. And the cytotoxicity of AAT (10 μg/mL) was detected at different time points (0, 4, 8, 12, 24, 48 h).

Gene transfection efficiency of AAT was evaluated in ATDC5 cells. Formulated with Cy3 labeled negative control RNA (25 nmol/L) and AAT (5 µg/mL), NgRNA/AAT was added into the ATDC5 cell culture medium, and incubated at 37 °C under 5% CO2 for 24 h. Photos were collected to verify whether AAT has the gene transfection ability using commercial transfection agent Lipofectamine 2000 (cat. No.11668019, Thermo) as control.

### Functional detection of miR365 antagomir/NPs* in vitro*

MiR365 antagomir/NPs was respectively added into primary chondrocytes and ATDC5 cell culture medium and incubated at 37 °C under 5% CO_2_ for 24 h. Commercial transfection agent lipo2000 was used to transfected miR365 antagomir as control. The expression of miR365 was detected by real-time PCR method. MiRNA quantification was determined by using Bulge-loopTM miRNA qRT-PCR Primer Set (one RT primer and a pair of qPCR primers for each set, Business secrecy) specific for miR365, designed and synthesized by RiboBio (RiboBio, Guangzhou, China). The U6 gene was used as an internal control. The qPCR reactions were performed on a Bio-Rad CFX96 Real-Time Detection System using SYBR Green Supermix kit (Bio-Rad) and data analysis were performed by using the 2^-ΔΔCt^ method.

### Preparation of fluorescently labeled yeast cell particle

*S.cerevisiae* SAF-Mannan, (SAF Agri, Milwaukee, WI) was used for yeast cell wall particle (YCWP) preparation as described previously 57 with some improvement. In brief, *S.cerevisiae* SAF-Mannan was alkaline-and-acid-extracted with pH 12.5 NaOH at 60 °C for 1 h and pH 4 HCl at 55 °C for 1 h. The product in the previous step was then extracted with isopropanol and acetone to get YCWP. YCWP was fluorescently labeled with 5-(4,6-dichlorotriazinyl) aminofluorescein (Thermo; 1 mg/mL in DMSO) to construct green gYCWP as described by Myriam et al 31. All the other chemical reagents used here were purchased from Sigma. Then the green labeled gYCWP were harvested and stored at -20 °C before use. More detailed steps can be found in the supplementary materials.

### Preparation of NPs-gYCWP

Diluted 10^6^ empty gYCWP into sterile saline and incubated with 2 µL EndoPorter (Gene Tools) for 1 h at 25 °C. Next, 10 µg AAT and 2 µL of 25 µM Cy3 red labeled negative control RNA (NgRNA) was used to construct NgRNA/NPs according to the method we described earlier. Then NgRNA/NPs and gYCWP were fully mixed and incubated at 25 °C in dark for 2 h to get NgRNA/NPs-gYCWP. Charged nanoparticles can be packaged into YCWP via electrostatic self-deposition.

During the preparation of NgRNA/NPs, we also added 10 µg AAT and 2 µL of 25 µM NgRNA into gYCWP one by one. Then the mixture was incubated at 25 °C in dark for 2 h to get NgRNA-AAT-gYCWP. The only difference between NgRNA/NPs-gYCWP and NgRNA-AAT-gYCWP was that we did not pre-assemble NgRNA and AAT into NPs (only AAT and RNA mixture) during NgRNA-AAT-gYCWP preparation. Confocal microscopy (Leica TCS SP8 STED 3X) was used to scan the construction of NgRNA/NPs-gYCWP and NgRNA-AAT-gYCWP.

### Functional detection of NPs-YCWP in macrophages* in vitro*

To verify whether NPs-YCWP system can be successfully recognized and engulfed by macrophages. RAW264.7 macrophages were cultured for 24 h in DMEM (ATCC, 30-2002) containing 10% FBS supplemented with 100 ng/mL lipopolysaccharide (LPS, Sigma-Aldrich, L2880). Formulated from Cy3 red labeled NgRNA/NPs and green labeled gYCWP, NgRNA/NPs-gYCWP was added into the LPS induced RAW264.7 macrophages culture medium, and incubated at 37 °C under 5% CO_2_ for 4 h. Photos were collected to verify whether NPs-YCWP system could be recognized and engulfed by macrophages. Then 10^5^/6-well YCWP containing 50 pmol miR365 antagomir/NPs (or negative control NgRNA/NPs) were added into the cell culture medium for 24 h. Cells were harvested by washing with phosphate buffered saline (PBS) before cell lysis for RNA isolation. Then gene expression of miR365, IL-1β, IL-6, Nr1D2 and TNF-α were quantified by RT-qPCR (Primers were shown in Table S1).

### Evaluation of the biological property of NPs-YCWP in simulated gastrointestinal fluid *in vitro*

Simulated gastric fluid (SGF) and simulated intestinal fluid (SIF) that was prepared according to United States Pharmacopeia specifications 32 were used to assess the stability of YCWP. Detailed steps were shown in the supplementary materials.

### Mice and diets

Aged eight weeks male C57BL/6 mice were purchased from the Animal Breeding and Research Centre of Xi'an Jiaotong University, China. They were housed under standard conditions of room temperature and dark-light cycles with plenty of food and water. All mice were treated according to the policy and regulations of the Institutional Animal Care and Use Committee. PTOA model mice were generated by surgical destabilization of the medial meniscus (DMM) as previously described 58. Two weeks after surgery, mice were randomly allocated to two groups. Control group was treated with negative control antagomir/NPs-YCWP (Ng) and the experimental group was treated with miR365 antagomir/NPs-YCWP (Exp). Each mouse was given 10^6^/YCWP with 100 pmol miR365 antagomir (or negative control antagomir) every other day. After 50 days, serum samples were collected from the tail veins for cytokines analysis via an enzyme linked immunosorbent assay (ELISA) and some organs were isolated for gene expression detection by real-time PCR. Samples of the small intestine and knee joints were collected for histological analysis.

### Toxicity and drug delivery ability of NPs-YCWP *in vivo*

Before analysing the PTOA therapy capacity, we evaluated the *in vivo* tissue toxicity of miR365 antagomir/NPs-YCWP. Mice were orally administrated with miR365 antagomir/NPs-YCWP or NgmiRNA/NPs-YCWP (10^6^/YCWP with 100 pmol miR365antagomir or NgmiRNA) every two days for 50 days. Then liver, lung and spleen of mice were collected for hematoxylin-eosin (HE) staining.

According to NPs-YCWP construction method, Cy5 fluorescent labeled RNA to construct Cy5RNA/NPs-YCWP. Mice were orally administrated with Cy5RNA/NPs-YCWP (10^6^ YCWP with 1nmol Cy5RNA) every 10 h for 3 times. The fluorescence signals of organs such as heart, liver, spleen, lung and kidney, brain, stomach and leg were detected by PerkinElmer (PerkinElmer IVIS Lumina XR Series III).

### Tissue macrophage isolation and gene expression quantification

Cells from small intestine, lung, spleen, bone marrow, spermatophore, testis, kidney and liver were collected for RT-qPCR detection. Before cell lysis for RNA isolation, all tissues need to be digested into a single cell suspension with different methods. For small intestine digestion, we used the method we described previously 23. We used 2 mg/mL collagenase D (Roche) solution for lung, spleen and testis digestion and 2 mg/mL collagenase IV (Sigma) for liver, kidney, spermatophore and bone marrow digestion. Detailed steps were shown in the supplementary materials.

For macrophages separation (macrophages from lung, spleen, small intestine and bone marrow), 400 µL/10^8^ cells buffer (PBS with 0.5% BSA, 2 mM EDTA) was used to suspend cells. All buffer need to be pre-cooled before use. CD11b MicroBeads (cat. No. 130-049-601, MiltenyiBiotec, Germany) were used for sorting macrophages according to the cell sorting manufacturer's instructions. Each cell suspension was divided into two, one for microRNA extraction and the other for total RNA extraction. The expression of miRNA 365, IL-1β, IL-6, Nr1D2, Line-1, TNF-α and arginase-1 were detected by real-time PCR (Primers were shown in Table S1).

### ELISA assay

Serum samples were collected from the tail veins for enzyme linked immunosorbent assay (ELISA) to estimate interleukin-1β (cat. No. 900-M47, PeproTech, USA), IL-6 (cat. No. 900-M50, PeproTech, USA), tumor necrosis factor alpha (TNF-α, cat. No. MTA00B, R&D Systems, USA), transforming growth factor-β (TGF-β, cat. No. MB100B, R&D Systems, USA) and granulocyte-macrophage colony stimulating factor (GM-CSF, cat. No. 900-M55, PeproTech, USA) according to the manufacturer's instructions. Three repeats of detection of each cytokine were performed and the signal was detected by Thermo Fisher Varioskan Flash (Thermo, Waltham, MA, USA).

### Histology, immunohistochemistry and immunofluorescence detection

After 50 days of oral administration, samples of small intestine and knee joints were collected for histological analysis. All samples were fixed overnight in 4% paraformaldehyde, decalcified (knee joints only), dehydrated, and then embedded in paraffin. We performed histological analysis using Safranin O/Fast Green staining and graded articular cartilage degeneration using Glasson 59 introduced Osteoarthritis Research Society International (OARSI) criteria. The primary antibody MMP13 (cat. No. ab39012, Abcam, China) was used for joint immunohistochemical staining. Immunofluorescence staining was performed with IL-1β (cat. No. 12242, CST, China) or F4/80 (cat. No. ab100790, Abcam, China).

### Statistical analysis

All statistical results were presented as means ± SD. Results were analyzed using Prism version 7 (GraphPad Software Inc.) for Windows. An unpaired two-tailed Student's *t* test was used for comparisons between two groups. A p value of < 0.05 was considered statistically significant.

## Supplementary Material

Supplementary methods, figures and table.
