# Supplementary material for: Yeast Cell wall Particle mediated Nanotube-RNA delivery system loaded with miR365 Antagomir for Post-traumatic Osteoarthritis Therapy via Oral Route
Source: Theranostics. 2020 Jul 9;10(19):8479–93. doi: 10.7150/thno.46761 (PMC7392020; doi:10.7150/thno.46761)
Supplement: Supplementary file 1 — Supplementary methods, figures and table. [file thnov10p8479s1.pdf]

Yeast cell wall particle mediated nanotube-RNA delivery system loaded with miR365 antagomir for post-traumatic osteoarthritis therapy via oral route

Long Zhang[1]*, Hang Peng[2], Wan Zhang[2], Yankun Li[2], Liang Liu[3] and Tongtong Leng[1]

[1]*Frontier Institute of Science and Technology, Xi'an Jiaotong University, Xi'an, Shaanxi 710054, PR China*

[2]*Health Science Center of Xi'an Jiaotong University, Xi'an, Shaanxi 710061, PR China*

[3]*Department of Orthopaedics, The First Affiliated Hospital of Xi'an Jiaotong University, Xi'an, Shaanxi 710061, PR China*

* To whom correspondence should be directed

E-mail: zhlong990@stu.xjtu.edu.cn

Phone: +8602985324587

Gene binding ratio of RNA to AAT nanotubes

The miR365 antagomir/NPs nanomaterial was self-assembled with miR365 antagomir (RiboBio, Guangzhou, China) and AAT nanotubes with different volume ratios.

1. The fixed miRNA (1 μL, 25 uM miRNA) was incubated with different doses of AAT (0, 1, 2, 4, 6, 8, 10 and 20 μg, 1 mg/mL) to form the RNA/AAT complexes.
2. Then sonicate the RNA/AAT complexes with 100 amplitudes for 150 s with a sonicator (Q700, Qsonica, USA).
3. The binding ratio of miRNA to AAT was detected using 2% agarose gel assay.

Evaluate the release and stability of AAT/RNA with different mass heparin sodium salt

1. Heparin sodium salt (40 mg/mL) was used to evaluate the release and stability of RNA/AAT.
2. Add 1 μL of miRNA (25 uM) into 10 μL RNase-free water and fully mixed.
3. Then 5 μL of AAT (1 μg/μL) was added into the RNA mixture in the previous step and fully mixed.
4. Sonicate the RNA/AAT complexes with 100 amplitudes for 150 s.
5. The complexes solution with v/v ratio of 5:1 (AAT/miRNA) was incubated with different mass heparin sodium salt (0, 0.63, 1.25, 2.5, 5, 10, 20 mg/mL and naked miRNA as control) for 1 h at 37 ℃.
6. The release of gene was detected using agarose gel assay as previously described.

Preparation of YCWP

S.cerevisiae SAF-Mannan, (SAF Agri, Milwaukee, WI) was used for yeast cell wall particle (YCWP) preparation as described below:

1. YCWP were prepared by suspending *S.cerevisiae* SAF-Mannan (15 g) in 200 mL of water, and the pH was adjusted to 12.5 with 1 M NaOH and heating to 60 ℃ for 1 h. During this period, shake the flask gently every 20 min (avoid violent vibration) to evenly disperse the yeast in the solution.

2. The insoluble material containing the cell walls was recovered by centrifuging at 1700 × *g* for 5 min.
3. This material was then suspended in 200 mL of water, brought to pH 4 with HCl, and incubated at 55 ℃ for 1 h. And shake the flask gently every 20 min.
4. Repeat step 2 and wash the insoluble material once with 200 mL of water, twice with 200 mL of dehydrated isopropanol, and twice with 200 mL of acetone. After each washing step, centrifuge the insoluble material at 1700 × *g* for 5 min.
5. The resulting slurry was dried at room temperature to produce slightly off-white powder.

Preparation of fluorescently labeled gYCWP

YCWP was used for green fluorescence labeled gYCWP preparation as described below:

1. YCWP (1 g) was washed with 40 mL of sodium carbonate buffer (0.1 M, pH 9.2).
2. The YCWP was recovered by centrifuging at 1700 × *g* for 5 min and resuspended in 90 mL carbonate buffer.
3. 10 mL of 5-(4,6-dichlorotriazinyl) aminofluorescein (Invitrogen; 1 mg/mL in DMSO) was added to the buffered YCWP suspension (10% v/v) and mixed at 25 °C in the dark for 16 h.
4. 50 mL of Tris buffer (2 mM) was added and incubated for 15 min at 25 °C.
5. The insoluble material washed with sterile pyrogen-free water for 4 times. Centrifuged the insoluble material at 1700 × *g* for 5 min at 4 ℃.
6. The green gYCWP was then dehydrated with 40 mL of absolute ethanol and 40 mL of acetone onece respectively. After each washing step, centrifuge the gYCWP at 1700 × *g* for 5 min.
7. Dried the gYCWP in the dark at 25 °C.

Tissue cells digestion

All tissues (from small intestine, lung, spleen, bone marrow, spermatophore, testis, kidney and liver) need to be digested into a single cell suspension with different methods before cell lysis for RNA isolation. We used 2 mg/mL collagenase D (Roche) solution for lung, spleen and testis digestion and 2

mg/mL collagenase IV (Sigma) for liver, kidney, spermatophore and bone marrow digestion with the steps as follows:

1. Rinse the dirt and blood clots of the lung (and liver) with pre-cooling phosphate buffered saline (PBS).
2. Place isolated lung (and spleen, kidney, liver, testis) in the 6 cm petri-dish with sufficient collagenase solution to cover the bottom of the dish.
3. Inject 500 μL of collagenase solution per lung and liver (100 μL per spleen, testis and kidney) with a 0.5 mL syringe and then cut the tissue into 1-2 mm^3 size pieces.
4. Digestion was achieved by adding 25 mL collagenase solution and incubation at 37 ℃, 155 rpm for 60 min (all samples including spermatophore and bone marrow).
5. Digested cell suspension was filtered through a 70 μm sterile filter and cell was washed twice with PBS.

The stability of YCWP in SGF and SIF

Simulated gastric fluid (SGF, which contains 0.2 g NaCl and 0.32 g pepsin in 100 mL water, and the pH was adjusted to 1.2 with HCl) and simulated intestinal fluid (SIF, which contains 0.68 g KH_2PO_4 and 1 g trypin in 100 mL water, and the pH was adjusted to 6.8 with HCl) were used to assess the stability of YCWP as described below: 10^5 YCWP was added into 500 μL of SGF and incubated for 4 hours at 37 °C. YCWP was recovered by centrifuging at 1700 × *g* for 2 min. Then resuspended the YCWP with 500 μL SIF and incubated for 6 and 8 hours at 37 °C.

Detect the protective effect of NPs-YCWP on NPs in SGF

NPs-gYCWP with red labeled negative control RNA (Cy3 RNA) was used to detect the protective effect of NPs-YCWP on NPs (Cy3RNA/NPs) at a pH 1.2 environment. 10^5 YCWP was added into 500 μL of SGF, brought to pH 1.2 with HCl, and incubated in the dark for 4-8 h at 37 °C. Then fluorescence was observed with fluorescence microscope.

NPs-YCWP can effectively release NPs in the SIF

NPs-YCWP that contained 10^4 YCWP was incubated with 50 μL SIF for 2-6 h at 37 °C. Then equal volume of heparin sodium salt (5 mg/mL) was added and incubated for 1 h at 37 ℃. Centrifuge the mixture at 1700 × *g* for 2 min and collect the supernatant liquid. The RNA intensity was measured using an agarose gel retardation assay.

Stability of NPs (RNA/AAT) in SIF

NPs were self assembled with RNA (2 μL of 25 μM negative control antagomir) and AAT (10 μg), and incubated in 50 μL SIF for 4 or 8 h at 37 °C (naked RNA as control). Then added equal volume of heparin sodium salt (5 mg/mL) and incubated for 1 h at 37 ℃. The RNA intensity was measured using an agarose gel retardation assay.

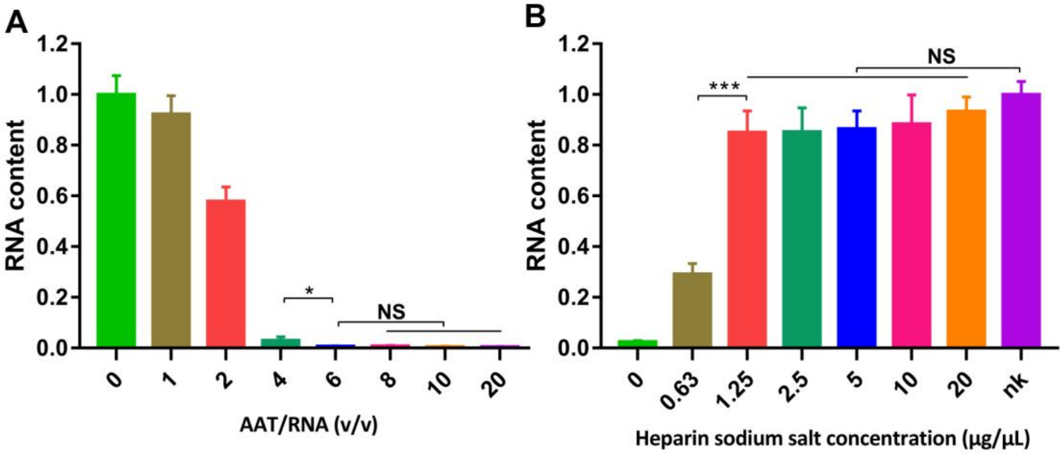

Figure S1. Quantification of the agarose gel electrophoresis results. (A) Quantification of agarose gel electrophoresis results of miR365 antagomir/NPs complexes at various v/v ratios (0, 1, 2, 4, 6, 8, 10 and 20). **(B)** Quantification of RNA release from miR365 antagomir/NPs by different mass heparin sodium salt (0, 0.63, 1.25, 2.5, 5, 10, 20 mg/mL). The nk means naked miRNA control. Data were expressed as mean ± SD. NS (no significance). **P* < 0.05, ****P* < 0.001 (n=3).

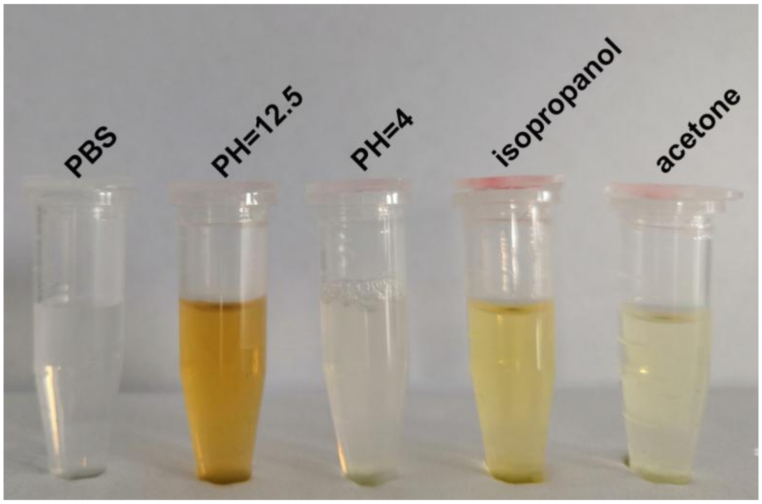

Figure S2.The eluate produced during the chemical treatment. *S.cerevisiae* SAF-Mannan was used for YCWP preparation by alkaline-and-acid extraction and then treatment with isopropanol and acetone. The eluate produced in each step during the chemical treatment was recorded.

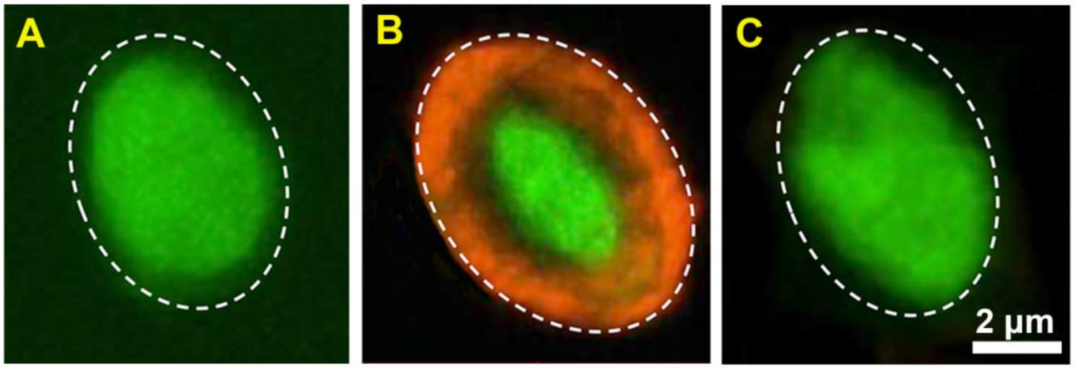

Figure S3. Confocal microscope results of NPs-gYCWP. YCWP was fluorescently labeled with aminofluorescein to construct green gYCWP. And AAT and Cy3 red labeled negative control RNA (NgRNA) was used to construct NgRNA/NPs. Then NgRNA/NPs and gYCWP were fully mixed to get NgRNA/NPs-gYCWP. Then gYCWP (**A**), NgRNA/NPs-gYCWP (**B**) and NgRNA-AAT-gYCWP (**C**) were observed under confocal microscope.

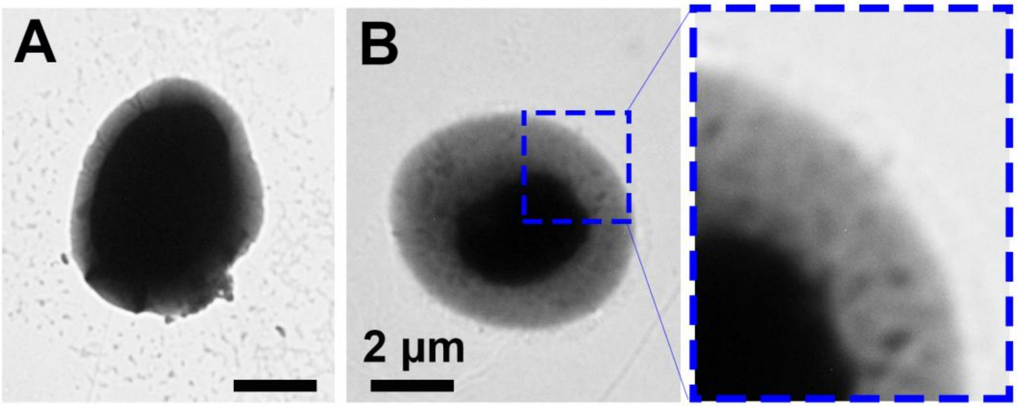

Figure S4. Transmission electron microscope results of YCWP and NPs-YCWP. *S.cerevisiae* SAF-Mannan was used for YCWP preparation by alkaline and acid extracted and treatment with alcohol and acetone to remove the genetic material of yeast itself. The NPs was self-assembled with miR365 antagomir and AAT nanotubes. Charged NPs can be packaged into YCWP via electrostatic self deposition. **(A)** YCWP. **(B)** NPs-YCWP.

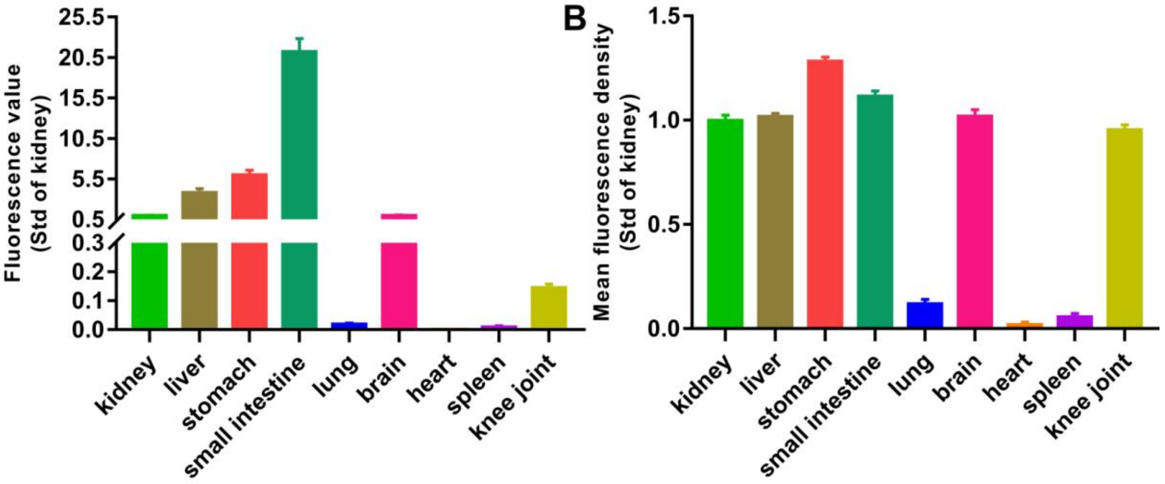

Figure S5. ***Ex vivo*** **semi-quantitative anslysis of fluorescence biodistribution. (A)** Fluorescence intensity in each organ was determined using Living Image software from PerkinElmer IVIS. **(B)** Mean fluorescence density in each organ. Data were expressed as mean ± SD (n=3).

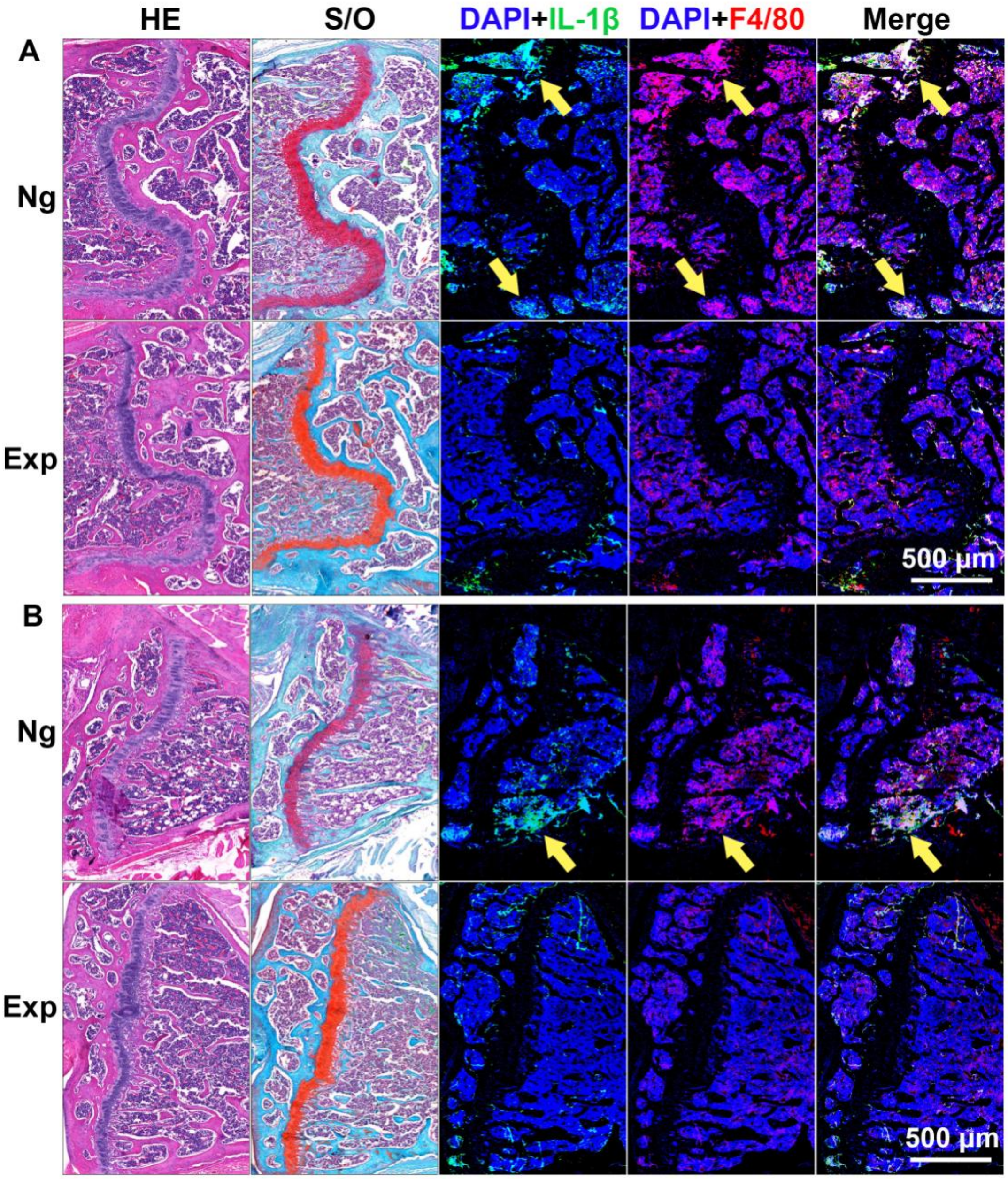

Figure S6. F4/80 and IL-1β double immunofluorescence in distal femur and proximal tibia of knee joint. Macrophages specific expression tag F4/80 and OA-related gene IL-1β was used to study the function of macrophages in PTOA knee joint. Compared with the Exp group, more F4/80 (red) and IL-1β (green) expression were detected both in distal femur (**A**) and proximal tibia (**B**). This is representative data of four repeats (n=4). The arrow pointing part indicated that F4/80 or IL-1β expression was high in this area.

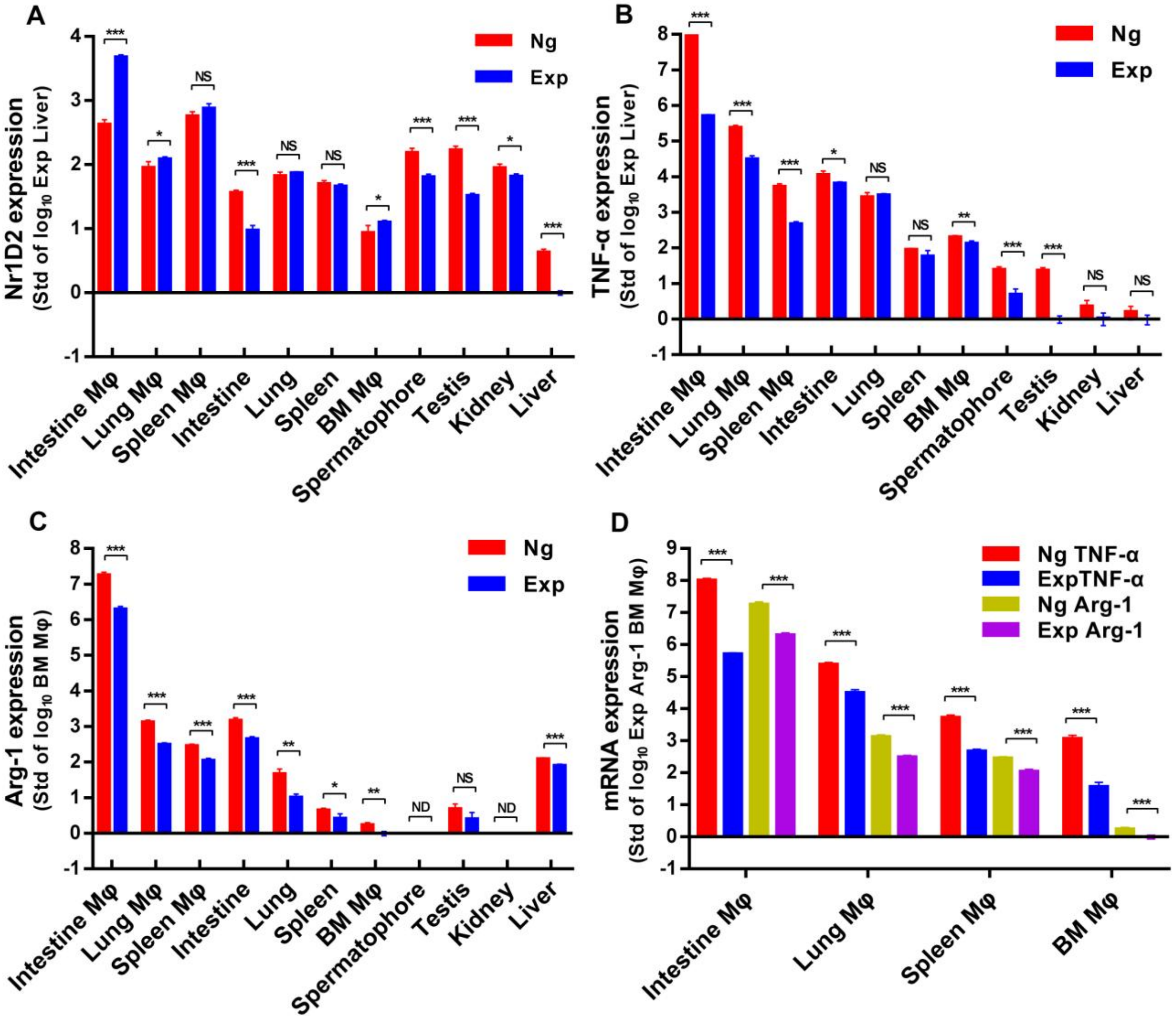

Figure S7. Expression of Nr1D2, TNF-α and Arg-1 in different cells *in vivo*. After oral administration of miR365 antagomir/NPs-YCWP (10^6/YCWP with 100 pmol miR365 antagomir) for 50 days, small intestine, lung, spleen, bone marrow, spermatophore, testis, kidney and liver were collected for gene expression quantification. CD11b MicroBeads were used for sorting macrophages (Mφ) from small intestine, lung, spleen and bone marrow. **(A)** Nr1D2, **(B)** TNF-α, **(C)** Arg-1 and summed of TNF-α and Arg-1 **(D)** expression were quantified by RT-qPCR. For each gene expression detect, we logarithmized the data and compared them to the smallest expressed tissue cells. Data were expressed as mean ± SD. NS (no significance), ND (Not detected). **P* < 0.05, ***P* < 0.01, ****P* < 0.001 versus control group (n=4).

Table S1. The information of miRNA and primers sequences

Name	Sequence (5' to 3')
miR365 mature sequence	UAAUGCCCCUAAAAAUCCUUAU
NgmiRNA mature sequence	UUUGUACUACACAAAAGUACUG
miR365 antagomir	AUAAGGAUUUUUAGGGGCAUUA
NgmiRNA	CAGUACUUUUGUGUAGUACAAA
18S forward primer	CGGCTACCACATCCAAGGAA
18S reverse primer	GCTGGAATTACCGCGGCT
IL-1β forward primer	AACCTGCTGGTGTGTGACGTTC
IL-1β reverse primer	CAGCACGAGGCTTTTTTGTTGT
IL-6 forward primer	TCCAGTTGCCTTCTTGGGAC
IL-6 reverse primer	GTACTCCAGAAGACCAGAGG
Nr1D2 forward primer	AGTGGCATGGTTCTACTGTGT
Nr1D2 reverse primer	GCTCCTCCGAAAGAAACCCTT
Line-1 forward primer	TGAGTGGAACACAACTTCTGC
Line-1 reverse primer	CAGGCAAGCTCTCTTCTTGC
TNFα forward primer	ACCCTCACACTCAGATCATCTTC
TNFα reverse primer	TGGTGGTTTGCTACGACGT
Arginase-1 forward primer	CTCCAAGCCAAAGTCCTTAGAG
Arginase-1 reverse primer	AGGAGCTGTCATTAGGGACATC
